# Differences in Attitudes toward Mental Health among Boys from Religious and Non-religious Families Experiencing Religious and Secular Education

**DOI:** 10.11621/pir.2022.0104

**Published:** 2022-03-30

**Authors:** Julia V. Borisenko, Elena V. Evseenkova

**Affiliations:** a Kemerovo State University, Kemerovo, Russia

**Keywords:** Mental health, attitudes, gender, personality development, orthodox families

## Abstract

**Background:**

Post-industrial society faces multiple stresses and developmental risks, both environmental and biological. The issues of mental health have become more dramatic and subject to debate. The current discourse about the religiosity-mental health nexus makes the study of differences in attitudes towards mental health among children from religious and non-religious families experiencing religious and secular education significant and relevant for practice.

**Objective:**

We studied the attitudes toward different spheres of life of children from Orthodox and non-religious families experiencing religious and secular education. We hypothesized differences in attitudes toward mental health by children from Orthodox families and non-religious families regardless of school choice. We expected the positive attitudes toward mental health to be greater for the children experiencing religious and secular education.

**Design:**

Our study assessed 340 primary school boys on a number of measures. The boys’ average age was 10.4 years old. The participants were divided into three groups, taking into consideration the family’s religiosity and educational characteristics.

**Results:**

The boys from Orthodox families had more positive attitudes toward family, life, people, their bodies, and their mental health than the boys from nonreligious families. These differences were also significant between groups of boys from religious and non-religious families experiencing secular education. The boys from religious families experiencing religious education had more positive attitudes toward their physical and mental health than the boys from religious families experiencing secular education.

**Conclusion:**

Positive attitudes toward both physical and mental health are more likely to be formed within religious families.

## Introduction

There are a large number of works supporting the idea of a religion-mental health nexus (Abdala, et al., 2021; Braam, et al., 2010; Wang, Al Zaben, Koenig, & Ding, 2021; Worthington & Langberg, 2012). Although the majority of these studies are focused on mental health issues, some authors have found statistically significant correlations between religiosity and physical health as well (Dutton, Madison, & Dunkel, 2018; Woodley of Menie & Fernandes, 2016).

Using the method of meta-analysis, H. Koenig (2012) concluded that religious belief and participation were positively associated with both physical and mental health, as well as with healthy behavior.

H. Koenig (2012) gives three possible reasons for these findings. The first is religion’s role as a specific conscious coping strategy. Religion provides genuine resources for coping with stress by giving eternal meaning to a person’s life. The second is religion’s pro-social doctrines. By following different religious pro-social guides, a person avoids stresses in social conflict, which helps reduce the risk of poor mental health. And the third is social support. The social dimension of religion builds a religious community that may provide a support network to help alleviate stress in both material and non-material ways (Koenig, 2012).

Besides this, the religiosity-health behavior nexus could be explained by religious teachings (using Biblical justifications) to avoid unhealthy behavior such as extensive alcohol use, drug use, or sexual promiscuity (McCree, Wingood, DiClemente, Davies, & Harrington, 2003; Myers, 2014; Wills, Gibbons, Gerrard, Murry, & Brody, 2003).

H. Koenig’s ideas are confirmed in findings that religious individuals use more effective coping strategies when dealing with stressful situations (Myers & Diener, 1995; Siegel, Anderman, & Schrimshaw, 2001). Religious people are considered more likely to help others, and to engage in more volunteering; they report fewer antisocial activities such as lying, cheating, and stealing (Schlenker, 2008). Also, greater moral commitment is associated with attitudes that signify greater psychological well-being, such as effective social functioning, greater internal control, sense of purpose in life, authenticity, and empathy (Schlenker, Chambers, & Le, 2012).

Various studies also report a strong association between genetic stability and religiosity. Thus, non-religiosity or beliefs in the paranormal are associated with a high mutational load (Dutton, Madison, & Dunkel, 2018; Koenig, McGue, Krueger, & Bouchard, 2005). And belief in the paranormal is associated with poor mental health (Dein, 2012; Schulter, & Papousek, 2008), schizophrenia (Schofield & Claridge, 2007), and manic depression (Thalbourne & French 1995). There is also a positive atheism-autism nexus (Woodley of Menie & Fernandes, 2016). So, since the parents and children share their genes, children from religious families and children from non-religious families may have differences in mental health.

Religiosity and moral or other attitude development originate in preschool childhood (Kashirskii, 2006; Kediarova, & Uvarova, 2012). D.V. Kashirskii believes that the first attitudes begin to appear at 6-7 years old, and are associated with the crisis of the seventh year (Kashirskii, 2006). These attitudes appear for the first time in personal communication with an adult at a child’s early age (Vygotsky, 1984). The meaning of any religious or moral concept is initially determined by the adult’s explanations of the concept to the child. Then, having discussed this meaning with the adult, the child forms his own sense of this concept, which can then be expressed to any other person as a moral attitude (Klochko, 2005).

So, we understand moral attitudes as a learned tendency to evaluate things in a certain way. Therefore, religiosity or any kind of spirituality (as well as non-religiosity) determines the formation of certain moral attitudes.

The moral attitudes and gender stratification in Orthodox families nowadays are more traditional than those of non-religious families (Mishhenko, 2016). Also, in a religious family, parental modeling is important for the development of personal attitudes (Francis & Casson, 2019). Commitment to tradition and conservatism are considered to be associated with some socially positive qualities, such being happier (Napier & Jost, 2008); being more helpful and generous (Brooks, 2006); working harder and having closer families (Schweizer, 2008); having greater personal agency and greater internal control, a positive outlook, transcendent moral beliefs, and generalized beliefs in fairness (Schlenker, 2012); and also with religiosity (Saroglou, Delpierre, & Dernelle, 2004). One of the key factors here is a sense of control, both personal (internal control, conscientiousness, hard work, perseverance, responsibility, and reliability) and over one’s life (by giving it eternal meaning and believing in a God who will ultimately look after a person (Dutton, Madison, and Dunkel, 2018)). It makes sense that religiosity would be closely related to life satisfaction (Myers & Diener, 1995) and mental health.

Thus, given the religion-health nexus, the issue of the impact of religious education becomes significant. The relationship between religious education on one hand, and personal development and mental health on the other, in both formal and non-formal educational settings, may be analyzed from two points of view. The first is how religious education affects personal development, attitude formation, and mental health. The second is how the values of the religious and non-religious worldviews can mediate the educational process. In the case of the children from Orthodox families studying in the Orthodox schools, it means that family, school, and public organizations are all involved in consistent interaction with the child, and work together to develop a consistent system of the child’s religious, moral, and gender attitudes (Boiazitova, 2011; Kediarova, 2013). So, as long as family influence is complemented by religious education (Razina, 2013), we would expect the personal attitudes of the boys from Orthodox families to differ from the attitudes of the boys from non-religious families.

Children from Orthodox families and non-religious families have different educational settings. The children from Orthodox families have different formal and non-formal religious educational opportunities available to them. Usually, they study in non-formal schools in their parish, which are organized by the clergy and the local laity. In the big cities, they also have an opportunity to study at Orthodox schools. The choice of the religious orientation of the school at first falls on the parents. In selecting the schools for their children, parents pursue their educational and developmental goals for them. Later, older children with different characteristics may self-select into different school systems. Under these circumstances, the school preference means a choice of their educational and communicational environment.

Orthodox schools give their students formal religious education as well as a basic secular education. There is a difference between Orthodox and secular public schools not only in the subjects taught (for instance, the Fundamentals of the Orthodox Religion) and disciplines’ content (when discussing the moral issues on literature or other subjects), but also in the personal influence of the teachers (who in the Orthodox schools are religious themselves, and some of them are even priests) on the development of students’ attitudes. This can explain religious education’s effects on the development of personal attitudes (for instance, attitudes toward education, people, work, family, life, and one’s physical and mental health) (Kashirskii, 2006). Positive attitudes toward people, family, and life may be an essential basis for a person’s capacity for non-conflictual communication (which reduces risks of communicational and emotional stress in conflicts).

Positive attitudes towards one’s body, and physical and mental health may determine healthy behavior, while students’ attitudes toward education and work may indicate social adaptation and may reflect back upon the educational process.

Thus, our study aimed to identify possible differences in the personal attitudes of the children from Orthodox families studying in Orthodox schools, and children from the non-religious families studying in the secular schools.

In this regard, we postulated the following research questions:

1. How do the moral attitudes of children from Orthodox families studying in Orthodox schools and the children from the non-religious families studying in secular schools differ?
2. Are there any differences in the moral attitudes of children from the Orthodox families and non-religious families studying in the secular schools?
3. Are there any differences in the moral attitudes of children from Orthodox families experiencing religious and secular education?
4. How could religious education be essential for a person’s mental health?

## Methods

### Participants

Our study was conducted in 2021 in the Russian regions of Kemerovo, Krasnoyarsk, Novosibirsk, and Tomsk. Since it was only a part of a more extensive study of the influence of family role distribution on children’s attitude formation, in the first stage we chose a sample of only boys. A total of 340 families participated in the survey, including 340 primary school boys: 120 boys from Orthodox families studying in an Orthodox primary school (the Orthodox gymnasium of Kemerovo city, the Orthodox gymnasium of Novokuznetsk city, and the Orthodox gymnasium of Novosibirsk city); 120 boys from non-religious families studying in secular schools; and 100 boys from Orthodox families studying in secular schools. The boys from the Orthodox schools had a religious component to their educational program, including studying such subjects as the Fundamentals of the Orthodox religion, Church singing, Church Slavonic language, etc. Their program also included regular participation in Church services.

The average age of the children was 10.4 years old. All the participants were living in Russia, and all were from urban families from the cities of Kemerovo, Novosibirsk, Tomsk, and Krasnoyarsk. All participants came from two-parent families, where 100% of the fathers were employed, as were 68% of the mothers (89% of mothers in the non-religious families and 58% of mothers in the religious families). The average number of children per family was 2.6 (SD = .89). All the respondents were recruited and compensated through the local research community.

### Procedure

A 15-minute, face-to-face interview with each child and at least one of the parents was conducted between January and April 2021. This gave us data on the nature of their responses and their demographic and family educational characteristics. All the participants obtained written permission from a parent, and had to answer several questionnaires.

At first, we compared the level characteristics of our sample with the statistical norms of children of that age. We concluded that our sample did not have considerable differences compared with the other children of the same age. Therefore, we could conduct further analysis of the results obtained from this sample of children.

Then we carried out the following pairwise comparisons: 1) boys from non-religious families experiencing secular education with boys from the Orthodox families experiencing religious education; 2) boys from non-religious families with boys from religious families, both experiencing secular education; and 3) boys from religious families experiencing secular education with ones from religious families experiencing religious education.

The F-Test for Equality of Two Variances at a significance level equal to 0.01 showed that the variances between all groups differed slightly. Also, all parameters were checked by the Kolmogorov Smirnov test and were found to conform to the normal distribution. We used the Student’s t-test and one-way ANOVA to find the differences between the groups of participants.

### Measures

*Family characteristics and characteristics of religious or non-religious education* were measured with a semi-structured interview specifically developed to study personal data such as age, gender, family, and educational characteristics.

*Masculine Gender attitudes* were measured using the questionnaire “Family Role distribution” (a = .63 for the composite) developed by Yu.E. Aleshina, L.Ya. Gozman, and E.M. Dubovskaya (1987). The children answered questions like “Who should determine the interests and hobbies of the family?;” “Who should have the most influence on the family mood?;” “Who in the family should care more about the comfort and convenience in the apartment?;” “Who in the family should play with the children?;” “Who in the family should do everyday shopping?;” “Whose work should be more important for the well-being of the family?;” and so on. Participants rated the items on a 4-point scale: “Dad = 1;” “Mostly dad, but sometimes mom too = 2;” “Mostly mom, but sometimes dad too = 3;” and “Mom = 4.”

*The Father’s Masculinity Appraisal* was studied through self-report using the Five-Item Self-Esteem scale based on the methodology of the self-esteem study developed by Dembo-Rubinstein (Ianshin, 2004). Participants were asked to name five qualities of an ideal person (a man and a woman), and rate the manifestation of those qualities in themselves and their fathers and mothers on a 5-point scale: “corresponds = 5;” “somewhat corresponds = 4;” “corresponds a little = 3;” “doesn’t correspond much = 2;” and “doesn’t correspond at all = 1.”

*Attitudes toward ethical standards* were measured via self-report using the method called the “Moral motivation measure” (a= .72), which was developed by the moral and ethical culture education laboratory at the Russian State Research Institute of Family and Education (Maliakova, 2005). The children were asked to imagine and choose their reaction in situations like “If one of your classmates cries, you a) will try to help him, b) will think about what might have happened, or c) will not care.” They were also asked how they would react to other situations such as “Someone whom you don’t know asks to play your game with you,” “Someone in the company is upset about losing the game,” and “Your classmate is offended by you.”

*Attitudes toward education, family, attitudes towards life, one’s body and physical health, personal responsibility, one’s mind, people, and work* were measured using the questionnaire of personal development provided by P.V. Stepanov, D.V. Grigoriev, and I.V. Stepanov (2003), which contains 49 statements (a =.77) about attitudes toward different spheres of life.

### Statistical analyses

Statistical analyses included calculation of the descriptive statistics, the Student’s t-test, and analysis of effect sizes (Cohen’s d).

## Results

Comparison of the boys from non-religious families experiencing secular education with the boys from the Orthodox families experiencing religious education using the Student’s t-test helped us measure the differences between these groups in gender attitudes; attitudes toward ethical standards; attitudes toward personal responsibility, and attitudes toward family, life, work, and people, as well as attitudes toward one’s body and physical health, and toward one’s mind and mental health protection (*Table 1*).

**Table 1:** Differences between boys from non-religious families experiencing secular education and boys from Orthodox families experiencing religious education

Attitudes	M(SD)**		p	**Cohen’s** *d*	Cohen’s U3
	Boys from non-religious families (secular education)	Boys from Orthodox families (religious education)			
Masculine attitudes * gender	1.86 (.22)	1.96 (.08)	.03	0.50	69.1
Father’s appraisal masculinity	3.79 (1.08)	4.01 (3.11)	.001	0.12	54.8
Positive ethical standards* attitudes toward	1.99 (1.38)	2.95 (1.16)	.005	0.57	71.6
Positive personal attitudes responsibility toward *	3.44 (.92)	4.38 (.86)	.005	0.64	79.3
Positive family * attitudes toward	10.68 (9.17)	15.14 (6.56)	.03	0.81	79.1
Positive life* attitudes toward	3.88 (7.62)	12.33 (5.82)	.005	0.51	69.5
Positive work * attitudes toward	4.75 (5.67)	12.43 (4.45)	.005	0.47	68.1
Positive education attitudes toward	11.02 (4.03)	12.04 (4.76)	.05	0.15	56.0
Positive people * attitudes toward	14.79 (6.42)	19.24 (4.88)	.01	0.71	76.1
Positive attitudes toward one’s body and physical health *	10.52 (8.22)	16.43 (7.39)	.01	0.92	82.1
Positive attitudes toward one’s mind and mental health protection*	9.42 (8.94)	15.67 (6.72)	.01	0.96	83.1

Note. * = significant differences; ** = standard deviations are in parentheses

One-way ANOVA undertaken to refine the hypothesis confirmed the results. Comparison of the indicators of the children’s gender attitudes showed that the children from religious families had more traditional gender attitudes. They evaluated themselves on gender qualities higher than the boys from non-religious families studying in secular schools.

**Figure 1.**
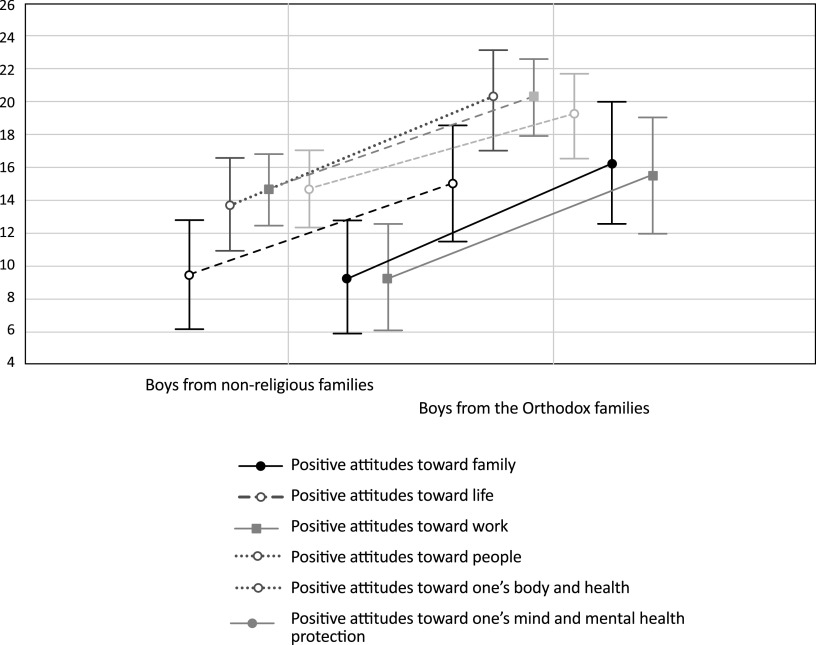
The results of one-way ANOVA for attitudes toward different spheres of life in boys from non-religious families experiencing secular education and boys from the Orthodox families experiencing religious education.

There were also significant differences between the children from religious and non-religious families in positive attitudes toward different spheres of life (*Figure 1*). The children from religious families demonstrated higher results on these scales.

In comparing the results of the children from religious (100 boys) and non-religious (120 boys) families studying in secular schools, we also found significant differences in masculine gender attitudes, attitudes toward ethical standards, personal responsibility, family, life, work, and people, and attitudes toward one’s body and physical health, and one’s mind and mental health protection (*Table 2*). The children from religious families also demonstrated higher results on these scales.

**Table 2:** Differences for boys from non-religious families and boys from Orthodox families experiencing secular education

Attitudes	M(SD)**		p	**Cohen’s** *d*	Cohen’s U3
	Boys from non-religious families (secular education)	Boys from Orthodox families (secular education)			
Masculine attitudes * gender	1.86 (.22)	1.91 (.09)	.01	0.45	67.4
Father’s appraisal* masculinity	3.79 (1.08)	4.21 (1.02)	.02	0.44	67.0
Positive ethical standards* attitudes toward	1.99 (1.38)	2.94 (.96)	.005	0.55	70.9
Positive personal attitudes responsibility toward *	3.44 (.92)	4.36 (1.06)	.005	0.58	71.9
Positive family * attitudes toward	10.68 (9.17)	15.07 (3.36)	.03	0.80	78.8
Positive life* attitudes toward	3.88 (7.62)	11.23 (4.72)	.005	0.54	70.5
Positive work * attitudes toward	4.75 (5.67)	11.83 (5.75)	.001	0.48	68.4
Positive education attitudes toward	11.02 (4.03)	11.87 (4.99)	.05	0.14	55.6
Positive people * attitudes toward	14.79 (6.42)	19.11 (6.17)	.02	0.69	75.5
Positive attitudes toward one’s body and physical health *	10.52 (8.22)	15.93 (5.89)	.02	0.91	81.9
Positive attitudes toward one’s mind and mental health protection*	9.42 (8.94)	14.27 (7.88)	.01	0.93	82.4

Note. * = significant differences; ** standard deviations are in parentheses

So, we can conclude that these attitudes are more likely to be formed in the family than in educational institutions. Then we compared children from the Orthodox families experiencing secular (100 boys) and religious (120 boys) education in school, and found significant differences only in their attitudes toward their bodies and physical health, and toward their minds and mental health protection; the positive parameters were higher among the boys studying in the Orthodox school (*Table 3*).

**Table 3:** Differences between boys from Orthodox families experiencing religious and secular education

Attitudes	M(SD)**		p	**Cohen’s** *d*	Cohen’s U3
	Boys from Orthodox families (secular education)	Boys from Orthodox families (religious education)			
Masculine attitudes gender	1.91 (.09)	1.96 (.08)	.05	0.03	51.2
Father’s appraisal masculinity	4.21 (1.02)	4.01 (3.11)	.05	0.09	53.6
Positive ethical standards attitudes toward	2.94 (.96)	2.95 (1.16)	.05	0.14	55.6
Positive personal attitudes responsibility toward	4.36 (1.06)	4.38 (.86)	.05	0.15	56.0
Positive family attitudes toward	15.07 (3.36)	15.14 (6.56)	.05	0.21	58.3
Positive life attitudes toward	11.23 (4.72)	12.33 (5.82)	.05	0.27	60.6
Positive work attitudes toward	11.83 (5.75)	12.43 (4.45)	.05	0.20	57.9
Positive education attitudes toward	11.87 (4.99)	12.04 (4.76)	.05	0.20	57.9
Positive people attitudes toward	19.11 (6.17)	19.24 (4.88)	.05	0.18	57.1
Positive attitudes toward one’s body and physical health *	15.93 (5.89)	16.43 (7.39)	.05	0.65	74.2
Positive attitudes towards one’s mind and mental health protection*	14.27 (7.88)	15.67 (6.72)	.02	0.69	75.5

Note. * = significant differences; ** standard deviations are in parentheses

## Discussion

Thus, among boys from religious families, attitudes toward different spheres of life differed from those of boys from non-religious families, which indicated a significant role of the family environment in the formation of child’s attitudes at primary school age. These results are comparable with the results of other studies in this field in Canada (Moore, Gomez-Garibello, Bosacki, & Talwar, 2016) and Britain (Francis & Casson, 2019; Francis, Fisher, Lankshear, & Eccles, 2018). Also, positive attitudes toward both physical and mental health, which are believed to be the essential parts of mental health (Jaberi, Momennasab, Yektatalab, Ebadi, & Cheraghi, 2019), were more likely to be formed at the Orthodox school than at the secular school. But as long as these parameters were still higher among boys from religious families, this finding requires further study, including children from religious families and religious schools of other confessions. Also, there is a need for surveying the effects of the religious educational environment on non-religious children.

Anyway, the main finding of this study is that religiousness is related to mental and physical health among children in Russia. Since the majority of the studies of the religiosity-mental health nexus have involved adults (Dutton, Madison, & Dunkel, 2018; Koenig, et al., 2015; Koenig, King, & Carson, 2012; McCree, et al., 2003; Myers, 2014; Napier & Jost, 2008; Wang, Zaben, Koenig, & Ding, 2021), our findings extend the theory by including children in the analysis.

The practical consequences of this study may lie in the field of psychological work on mental health issues with both individuals and families. Besides this, modern education aims to build a psychologically comfortable and safe educational environment for children. So, the ideas of moral development and its association with mental health outcomes may be helpful for educational practices.

Further studies of religious families’ influence on children’s attitudes and mental health, and investigation of the ways to build a favorable environment for the formation of attitudes towards mental and physical health in children of other ages using new approaches (Francis, Lankshear, & Eccles, 2021), will help to complement these results and create a holistic psychological basis for the practical work of psychologists dealing with education, parenting, and family problems, as well as help teachers and parents.

## Conclusion

Our study helped to determine the differences in attitudes towards different spheres of life (including mental and physical health) among children from religious and non-religious families. Children from Orthodox families had more positive attitudes towards mental and physical health than children from non-religious families, regardless of school choice. But for the children from religious families experiencing religious education, and non-religious families experiencing secular education, the differences were more tangible. So, we can conclude that positive attitudes towards both physical and mental health are more likely to be formed within religious families. But beyond that, children from religious families experiencing religious education scored higher in their attitudes toward mental and physical health than children from religious families experiencing secular education. We can interpret this as an indication that a religious educational environment strengthens positive attitudes toward both physical and mental health.

## Limitations

Some limitations of the current study should be mentioned. First, most measures for this study relied on the boys’ self-reports. In further studies, some parameters could be evaluated using reports by external observers, such as the parents.

Second, our study was focused on children from two-parent families living with their biological parents. The sample may have underrepresented boys from one-parent families. Children living in adopted families were also not represented. Third, our sample was represented only by boys. In our further study, we plan to explore gender differences, if any. Fourth, this study’s use of a convenience sample limits the generalizability of the results.

Finally, the study’s results were limited by the fact that we did not use genetic designs. We didn’t analyze twin samples and didn’t control for the participants’ sibling positions or the coincidence of siblings’ responses.

Despite these limitations, the current study furthers our understanding of the religion-mental health nexus. We need further study to answer some questions raised by our results. Some of them are: what the reasons of negative model of male behavior in children from non-religious families are; and what educational methods and types of psychological help could contribute to work with these children.
